# OHDSI Standardized Vocabularies—a large-scale centralized reference ontology for international data harmonization

**DOI:** 10.1093/jamia/ocad247

**Published:** 2024-01-04

**Authors:** Christian Reich, Anna Ostropolets, Patrick Ryan, Peter Rijnbeek, Martijn Schuemie, Alexander Davydov, Dmitry Dymshyts, George Hripcsak

**Affiliations:** Coordinating Center, Observational Health Data Sciences and Informatics, New York City NY 10032, United States; OHDSI Center at the Roux Institute, Northeastern University, Portland ME 04101, United States; Department of Medical Informatics, Erasmus University Medical Center, 3015 GD Rotterdam, The Netherlands; Coordinating Center, Observational Health Data Sciences and Informatics, New York City NY 10032, United States; Department of Biomedical Informatics, Columbia University Medical Center, New York City NY 10032, United States; Odysseus Data Services, Cambridge MA 02142, United States; Coordinating Center, Observational Health Data Sciences and Informatics, New York City NY 10032, United States; Department of Biomedical Informatics, Columbia University Medical Center, New York City NY 10032, United States; Observational Health Data Analytics, Janssen Research & Development, Titusville NJ 08560, United States; Coordinating Center, Observational Health Data Sciences and Informatics, New York City NY 10032, United States; Department of Medical Informatics, Erasmus University Medical Center, 3015 GD Rotterdam, The Netherlands; Coordinating Center, Observational Health Data Sciences and Informatics, New York City NY 10032, United States; Observational Health Data Analytics, Janssen Research & Development, Titusville NJ 08560, United States; Coordinating Center, Observational Health Data Sciences and Informatics, New York City NY 10032, United States; Odysseus Data Services, Cambridge MA 02142, United States; Coordinating Center, Observational Health Data Sciences and Informatics, New York City NY 10032, United States; Observational Health Data Analytics, Janssen Research & Development, Titusville NJ 08560, United States; Coordinating Center, Observational Health Data Sciences and Informatics, New York City NY 10032, United States; Department of Biomedical Informatics, Columbia University Medical Center, New York City NY 10032, United States

**Keywords:** OHDSI, controlled vocabulary, common data model, observational data

## Abstract

**Importance:**

The Observational Health Data Sciences and Informatics (OHDSI) is the largest distributed data network in the world encompassing more than 331 data sources with 2.1 billion patient records across 34 countries. It enables large-scale observational research through standardizing the data into a common data model (CDM) (Observational Medical Outcomes Partnership [OMOP] CDM) and requires a comprehensive, efficient, and reliable ontology system to support data harmonization.

**Materials and methods:**

We created the OHDSI Standardized Vocabularies—a common reference ontology mandatory to all data sites in the network. It comprises imported and *de novo*-generated ontologies containing concepts and relationships between them, and the praxis of converting the source data to the OMOP CDM based on these. It enables harmonization through assigned domains according to clinical categories, comprehensive coverage of entities within each domain, support for commonly used international coding schemes, and standardization of semantically equivalent concepts.

**Results:**

The OHDSI Standardized Vocabularies comprise over 10 million concepts from 136 vocabularies. They are used by hundreds of groups and several large data networks. More than 8600 users have performed 50 000 downloads of the system. This open-source resource has proven to address an impediment of large-scale observational research—the dependence on the context of source data representation. With that, it has enabled efficient phenotyping, covariate construction, patient-level prediction, population-level estimation, and standard reporting.

**Discussion and conclusion:**

OHDSI has made available a comprehensive, open vocabulary system that is unmatched in its ability to support global observational research. We encourage researchers to exploit it and contribute their use cases to this dynamic resource.

## Introduction

Population research involving observational data from electronic health records (EHR) and administrative claims requires a large scale to cover the uptake of new drugs or therapies and rare outcomes. Large sample size and diverse populations^1^ can provide sufficient statistical power to address infrequent conditions and improve the generalizability of findings.^2^ The scale can be achieved through central aggregation of data or through distributed data networks.^3^ While centralized systems provide better data retrieval performance and more efficient data mining, distributed data networks are gaining increasing traction for their scalability, flexible data access workflows,^4^ and protection of patient privacy.^1^

However, efficient analysis of data hidden behind firewalls or from multiple sources is very much simplified if those data are standardized to a common data model (CDM).^1^ Such standardization into an externally defined set of tables and relationships provides a common context to the clinical data elements, which is necessary to create unified analytical and quality assurance methods and algorithms that can run across the network. Consequently, major data networks such as Sentinel, PCORNet, or Observational Health Data Sciences and Informatics (OHDSI) each have adopted a CDM.^5^^,^^6^

Aside from standardizing the structure of the data, content harmonization is achieved through medical vocabularies, or coding schemes, which are maintained by various organizations and professional societies to ensure accurate and consistent communication about patient care and treatment. They can be simple sets of codes or terms to extensive hierarchies or ontologies, with often intersecting coverage of healthcare domains.^7^ For any given domain, members of distributed data networks may use different vocabularies, different versions of the same vocabulary, non-public vocabularies, or no vocabulary at all in their data.^8–11^

For distributed data networks that are confined to data from the United States, CDMs have been trying to get away without harmonization of the coding schemes by benefiting from the quasi-standardization achieved through US government billing rules.^12^^,^^13^ These are similarly adopted by private sector payers, and, to facilitate effective reimbursement, by EHR systems as well.^14^ However, those models degrade with each new or upgraded standard.^15^ Also, other countries established their own systems for representing diagnoses, procedures, and drugs,^16^ and standardization among them cannot be done without content harmonization.^17^ The latter can be achieved through an ad-hoc approach, where the coding of data is left unchanged and the harmonization effort is added to the analysis,^18^ or as part of a central reference model providing an a priori semantic standardization.

For its clinical research network, OHDSI chose the central system. The network is the largest in the world encompassing 331 data sources with 2.1 billion (partially duplicated) patient records across 34 countries^19^ connected through an open science collaborative and requiring each data partner to opt in for each research study. Some of them are networks themselves, such as All of US,^20^ eMERGE,^21^ EHDEN,^22^ and N3C.^23^

A central reference system needs to serve the main tasks of observational research: (1) cohort definition, (2) covariate construction, (3) large-scale analytics, and (4) result reporting, which are driving its requirements (Table 1).

**Table 1.** Requirement for an effective central reference ontology supporting the OHDSI Network.

Requirement	Definition
Standard concepts	Unique concepts of fully pre-coordinated medical entities, to be stated as fact, no negations of facts, no reference to the past, and no flavors of null (unknown, not reported, etc.)
Concept domains	Assignment of concepts to domain categories (condition, drug, visit, etc.)
Comprehensive coverage	In each domain, standard concepts must cover all possible entities and mappings from terms and codes used in databases around the world
Polyhierarchies	Precalculated hierarchies organizing concepts
Efficiency	Computationally efficient data model
Use case focus	Storing and analyzing patient-level data for evidence generation

The Unified Medical Language System (UMLS), the largest public resource integrating medical terminologies, was designed to support patient care, medical education, library service, and product development^24^ but has also found application in artificial intelligence, data mining, and knowledge discovery.^25^ Such wide appeal creates complexity and content unrelated to our use cases, making it unsuitable to serve as a distributed reference system directly. Instead, we built a dedicated solution called the OHDSI Standardized Vocabularies. In this article, we describe its design, generation, quality assurance, and distribution as well as the challenges associated with its creation.

## Methods

We created the system of the OHDSI Standardized Vocabularies as an ontology serving the Observational Medical Outcomes Partnership (OMOP) CDM, a relational database model for the representation of patient data.^26^ To achieve a fully normalized model and to obviate the need for open text fields, we are maintaining a network-wide common reference system and are making it available to the end users who store it in tables of their relational database. We designed the system so that it preserves the original meaning of each record and transforms it to a common representation for analytical methods.

### Vocabularies and concepts

The OHDSI Standardized Vocabularies is a collection of public standard vocabularies used in the network, which we consolidate from their different original formats and life-cycle conventions into the CDM table structure. This staging process involves assigning stable identifiers to individual codes, which are unique across the entire system, adding additional attributes and establishing the relationships to integrate the vocabularies into an overall ontological structure. For internal reference and for purposes of semantic standardization, we also author our own vocabularies and relationships.

After staging, the individual elements or codes of the vocabularies are called concepts. Even though each concept has a name (description) and any number of synonyms, we make no attempt at comprehensive lexical coverage to support natural language processing or information retrieval. All concept names are in English, synonyms can be of any language. They, together with the relationships, form the framework of the ontology.

### Domains

**Figure 1.**
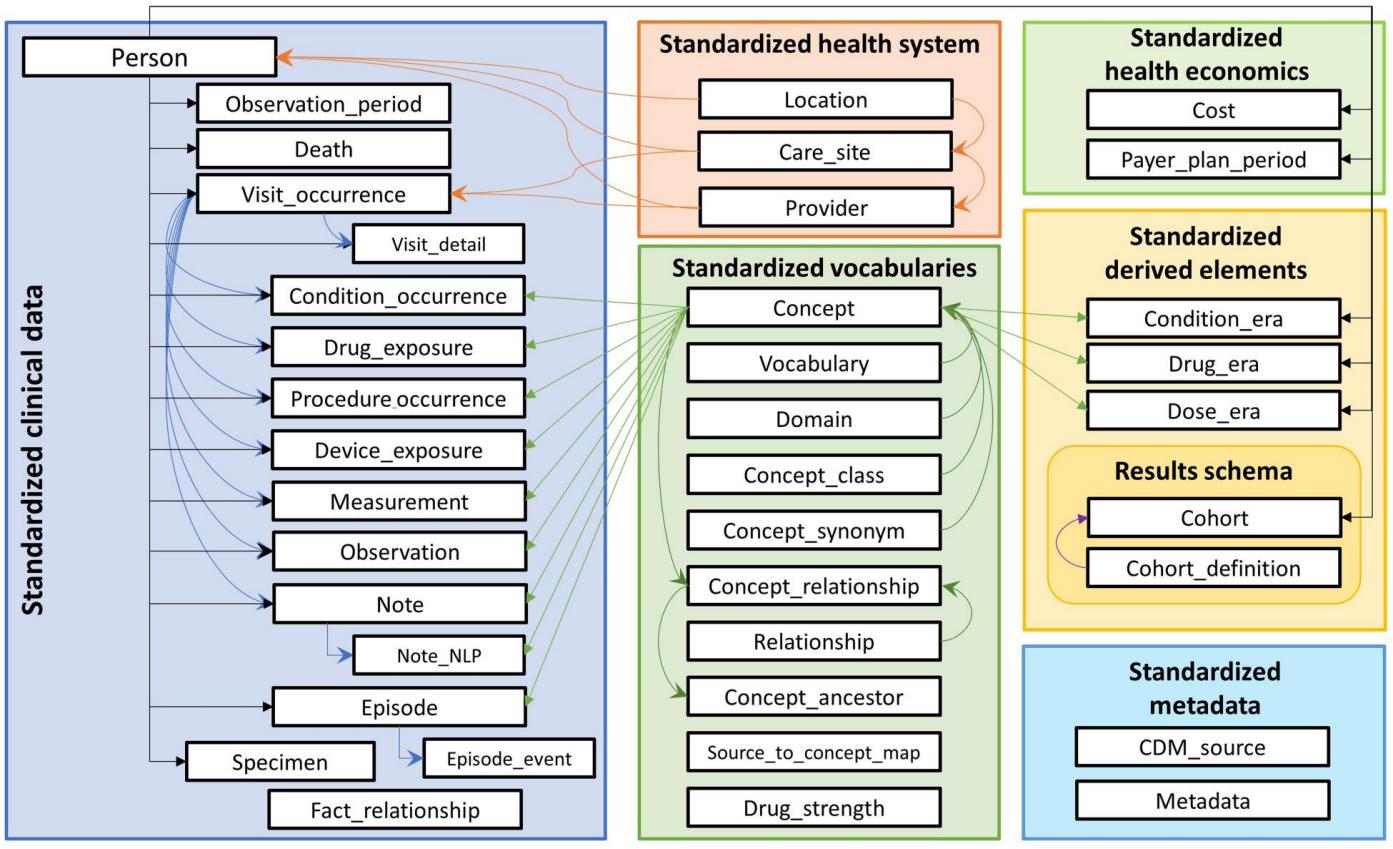
Overview of structure of OMOP CDM and Standardized Vocabularies. Grey arrows indicate foreign key relationships, and orange arrows indicate relationships of concepts, which follow domain-field association.

We assign a semantic category to each concept, called a domain. Each domain corresponds to a specific field in the OMOP CDM, which contains a clinical fact. For example, the condition_concept_id field in the CONDITION_OCCURRENCE table is reserved for concepts with the “Condition” domain. Other domains are Procedure, Drug, Device, Visit, Observation, Measurement, Race, Gender, Cost, etc., with their respective database fields. The assignment follows the domain definition laid out in the documentation^26^ of the CDM fields (Figure 1). This approach ensures that the content is correctly stratified according to the model, rather than by the choice of the vocabulary makers, and any record about, say, a procedure will be only recorded in the corresponding procedure_concept_id field. This drastically simplifies data analysis and makes the model independent of the choice of vocabularies. It also means that one vocabulary might have concepts from more than one domain. For example, concepts of the CPT-4, even though its name suggests containing only procedure concepts, can also belong to the Device (such as 77334 “Treatment devices, design and construction; complex irregular blocks, special shields, compensators, wedges, molds or casts”), Drug (90690 “Typhoid vaccine, live, oral”), Measurement (85045 “Blood count; reticulocyte, automated”), Visit (1021885 “Birthing Center”), or Observation (2016F “Asthma risk assessed”) domain. Some vocabularies come with their own semantic categories, which we store in a separate field (concept_class_id) and which may resemble domains. For example, SNOMED-CT stratifies concepts into body structure, clinical finding, environment/location, organism, procedure, etc., but these are not connected to the domain heuristic applied in the Standardized Vocabularies.

### Standardization of concepts

Following the closed-world model of observational data, we must build domains with the goal of complete coverage of the semantic space. For example, the Procedure domain should contain any diagnostic and therapeutic procedure carried out on patients. For some domains, existing vocabularies come close to this requirement and become preferred sources. For example, SNOMED-CT’s 112 118 Condition domain concepts contain all but the most exotic diseases and conditions that can be diagnosed in a patient, except for detailed cancer diagnoses supplied by ICD-O-3. For other domains, such as Procedure, we populate the domain through a union of concepts from various vocabularies. In the Drug domain, we have the situation that, like SNOMED-CT in Condition, the vocabulary RxNorm represents very well the pharmaceutical market of the USA, but no publicly available vocabulary does an adequate job for products marketed in other countries. We therefore constructed a new RxNorm Extension vocabulary to fill this gap.

Placing concepts from different vocabularies into a single domain inevitably creates redundancies, that is, several concepts with a similar or identical meaning. To avoid having to choose from an ambiguous set of similar concepts and the burden on the analyst having to request all alternatives in data retrieval queries, we created a heuristic to elevate one concept to be the main representative, called the standard concept. Only standard concepts are allowed to be used to represent facts in OMOP CDM tables. The other concepts carrying that same meaning are called source concepts and a separate field stores the concept used in the source data. For example, the concept derived from SNOMED-CT 49436004 “Atrial fibrillation” is the standard concept for representing this condition, while similarly named source concepts 427.31 from ICD-9-CM, I48.91 from ICD-10-CM, G573000 from Read, D001281 from MeSH and 10003658, 10051363, 10001452, 10003796, 10016566, and 10066582 from MedDRA, any of which may have been used in the original data, are not.

The closed-world assumption means that all entities and facts and their timing are known. This prohibits standard concepts from defining negative facts or projecting them to another time. For example, the concept taken from Read 1951.00 “No indigestion” cannot adopt standard designation, as the absence of that condition is simply signified by the absence of a record of “indigestion.” Neither can ICD-10 I25.2 “Old myocardial infarction” get such status as the condition happened in the past. To capture facts lacking timing, we use a standard Observation concept “History of” with the fact as value. For all source concepts where no standard concept can be assigned, a special concept with the id = 0 represents a generic unknown or undefined fact in any domain.

**Figure 2.**
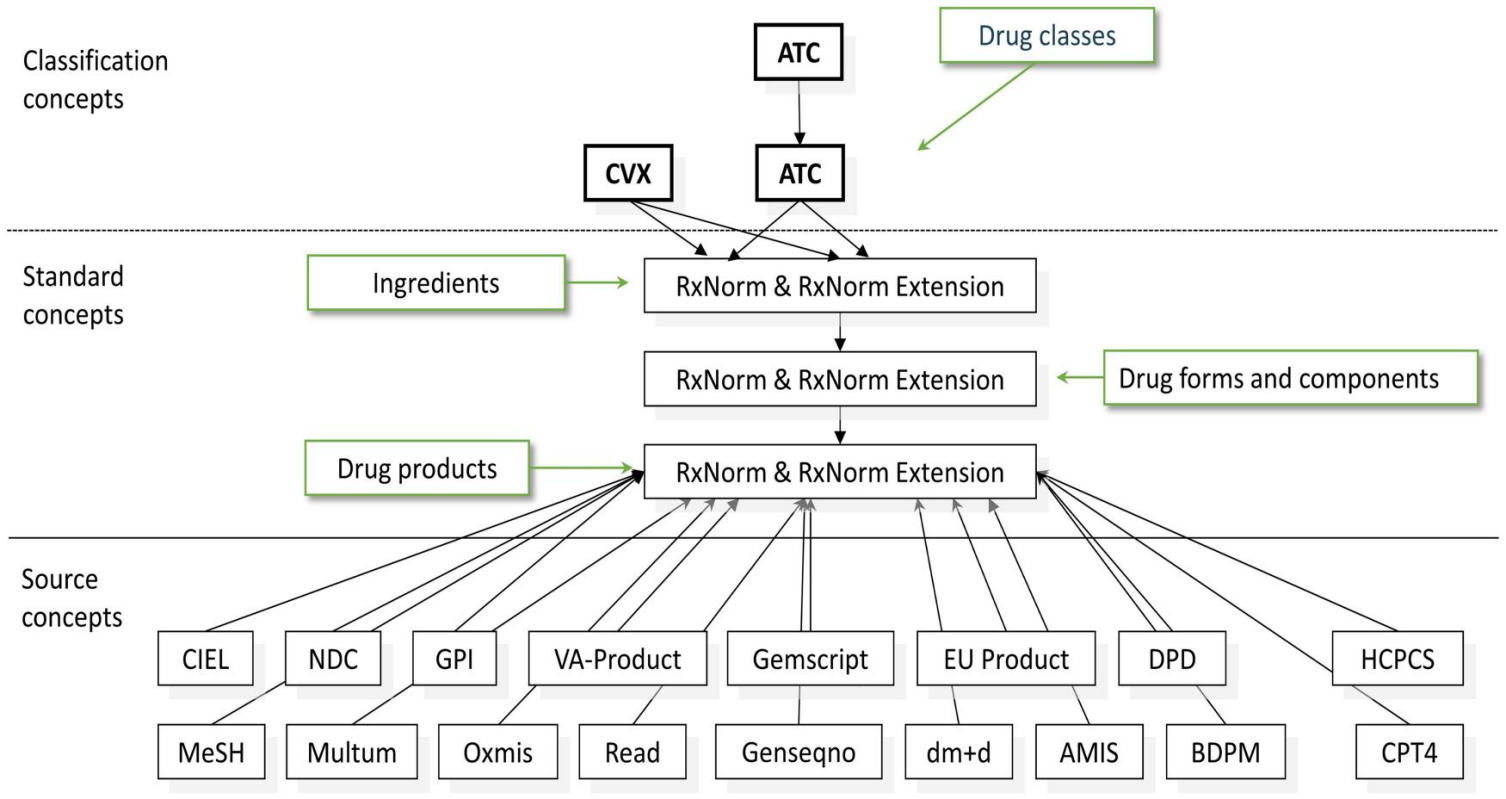
Different types of concepts of the OHDSI Standardized Vocabularies and the vocabularies they are derived from in the Drug domain, and their hierarchical system. Arrows designate hierarchical and “Maps to” relationships.

A third class of concepts not used to record distinct clinical facts and generally used for reporting and analysis are classification concepts. ATC S01BC “Antiinflammatory agents, non-steroids” is an example for such a classification concept, while an individual drug product such as RxNorm 198440 “Acetaminophen 500 mg Oral Tablet” is a standard concept, connected to the classification concept through the hierarchy (Figure 2).

### Mapping, hierarchical, and other relationships between concepts

We achieve semantic standardization by selecting one referent concept per meaning, the standard concept. We map the remaining non-standard source concepts to standard ones as a service to the OHDSI community. Source concepts without clear semantic content or outside the realm of observational research are not mapped. We adopt mappings directly from the sources or indirectly from the UMLS, or create them *de novo*. For successful standardization, we aim at comprehensive coverage of all source concepts, which is a substantial task of importing, reviewing, modifying, and validating the maps. They are distributed together with the concepts as part of the OHDSI Standardized Vocabularies to achieve consistency across data sites.

We connect standard and classification concepts through polyhierarchies, defined as hierarchical trees allowing for more than one parent per concept.^10^ Non-standard concepts are not included in hierarchies, even though they may have a hierarchy in their source vocabulary. For example, ICD-10 concepts being all non-standard come with a simple hierarchy, which is not included in our polyhierarchy. On the other hand, standard SNOMED-CT concepts already have an internal hierarchy, to which we append ICD-O-3 concepts, forming a common hierarchical structure for the Condition domain. Like with mapping relationships, we aim at building a comprehensive hierarchical structure, which requires substantial generation, review, and validation of hierarchical relationships. The entire hierarchical structure is pre-computed, combining all concepts, Isa/Subsumes relationships and lateral relationships linking concepts from different vocabularies, and placed into a separate table.

Non-hierarchical (eg, “part-of,” “Has pathology,” and “Using device”) relationships are not curated by OHDSI but may be imported if available from the source vocabulary for convenience. We make no attempt to create a comprehensive semantic knowledge base of non-mapping or hierarchical relationships between concepts.

### Life cycle and distribution

The OHDSI Standardized Vocabularies are made available as a free, open-source system driven, and maintained by a dedicated team in the OHDSI community. It requires ongoing maintenance, the result of which we distribute through regular releases. We create these using a partially automated system^27^ and place them into the online browsing and download system ATHENA.^28^ Vocabulary releases happen semiannually or triggered by urgent community requests.

Standard concepts are always included in the download, while classification and source concepts need to be requested. For vocabularies not in the Public Domain, a distribution license must be obtained from the authoring organization or through the UMLS.

The OMOP CDM is a model for longitudinal patient data, which means it needs to support concepts that were used in the past and might no longer be active. It also needs to respond quickly by adding new concepts and placing them into context. If concepts are dropped from their source vocabulary, they are not removed, but assign an end date and a flag (invalid reason), reflecting their status. Similarly, within and across-vocabulary relationships can become invalid or updated, which is reflected in the same fashion.

In general, codes are not reused in their source vocabularies. But there are exceptions to this rule, in particular for HCPCS, NDC, and DRG codes. We assign separate concepts to each with a unique concept identifier, a validity date range, and an invalid flag, except for the latest of them.

### Quality assurance

For each release, we apply a multi-stage quality assurance (QA) process, using automated and manual components. It ensures (1) conformance with the database model including referential integrity and enforcement of constraints; (2) integrity rules for domains, concept classes, vocabulary IDs, and relationships as well as consistency of validity dates and validity status; and (3) semantic QA examining vocabulary alignment. At this stage, non-standard concepts are checked for potential standard mapping as well as the polyhierarchies are examined for consistency. We also run a community complaint capture and resolution system.

## Results

The OHDSI distributed data network, which uses the OHDSI Standardized Vocabularies as a central semantic reference system, has seen massive uptake since its inception in 2009 (initially as the OMOP Standardized Vocabularies).^29^

### Overall content

**Figure 3.**
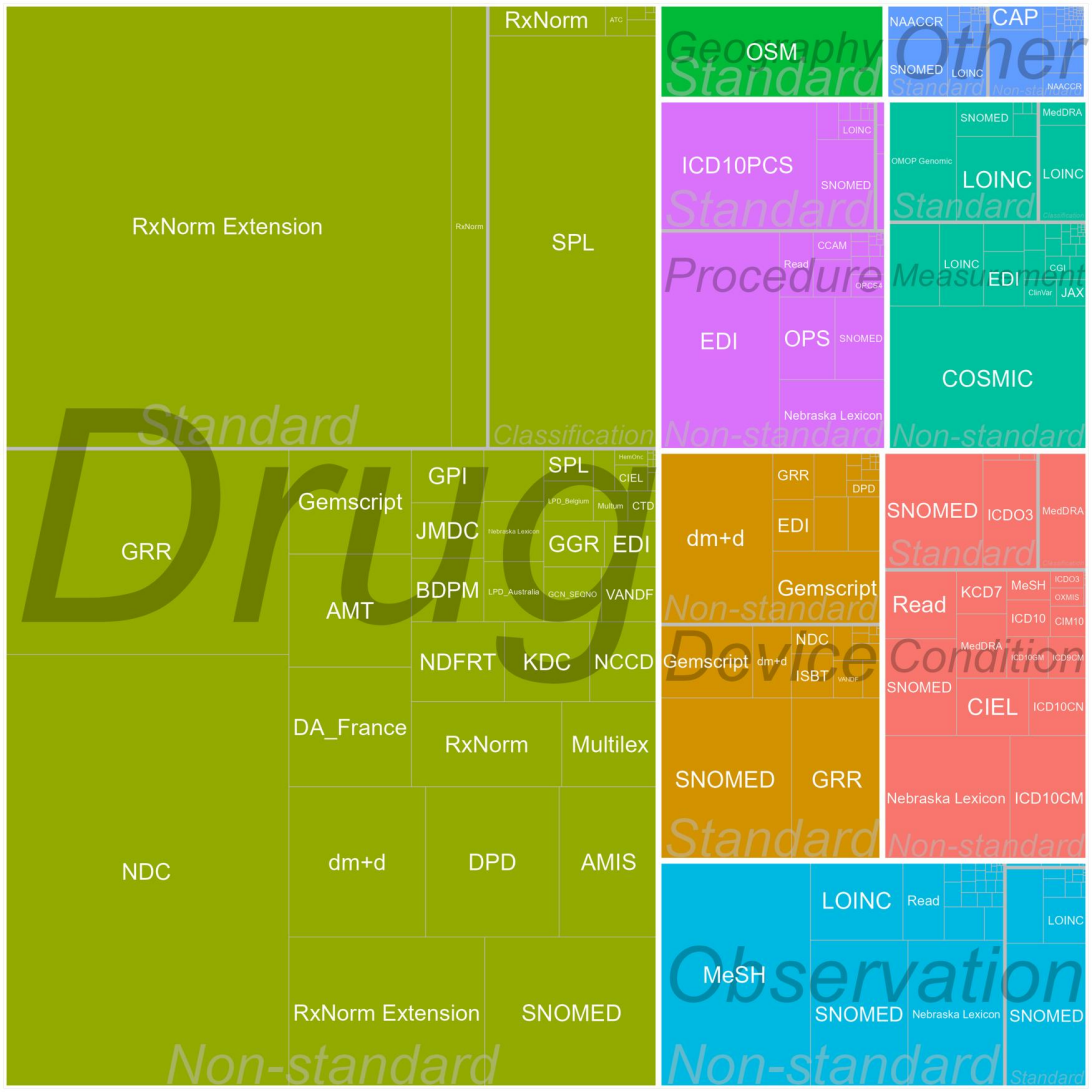
Distribution of the concepts in the OHDSI Standardized Vocabularies organized by domain (color) and vocabularies (boxes sized by the number of concepts), standard and non-standard.

As of March 2023, the vocabularies comprise 8 761 976 valid concepts (10 574 359 total) from 136 vocabularies, 101 of which are incorporated from external sources (Table S1). The single largest source is the UMLS, supporting 15 vocabularies and their relationships. Figure 3 shows the composition of vocabularies stratified by OMOP domain.

Since the construction of the Standardized Vocabularies started from OMOP, a project focused on drug surveillance for the United States, many of the vocabularies are of American origin.^29^ However, the composition is increasingly becoming international (Table S1).

### Concepts and relationships

Standard concepts are assigned from some preferred vocabularies (Table S2), for example, SNOMED-CT and LOINC for laboratory tests and vital signs; CPT-4, SNOMED-CT, and ICD-10-PCS for diagnostic and treatment procedures; and RxNorm, RxNorm Extension, and CVX for drugs. Standard concepts account for 40.5% of the total (3 550 260 out of 8 761 976 valid concepts). Source concepts are predominantly coming from the source vocabularies of many patient databases, such as NDC, ICD-9-CM, ICD-10, Read, dm+d, and Multilex. In total, non-standard concepts account for 50.1% of the content (4 389 657 valid concepts). Classification concepts mostly exist in the Drug and Measurements domains and make up for the remaining 9.4% (822 059). A breakdown of these concept types in the main 6 domains is provided in Table S3.

There are more than 28 million valid relationships between concepts, both within vocabulary and across vocabularies. Relationships always exist twice, one in each direction. The most common type of relationship is hierarchical (Isa/Subsumes), accounting for 38.3% of all relationships. It is followed by mapping relationships at 14.1%, mapping 66.8% of the source concepts to standard ones. Most of the other relationships belong to RxNorm and RxNorm Extension defining drugs and their components (Table S4).

Mapping relationships are not necessarily exclusive between one source and one standard concept (Table 2). The most common ones are many-to-one, as concepts from multiple source vocabularies map to the same standard concept. For example, SNOMED-CT 94899001 “Neoplasm of uncertain behavior of larynx” is a standard concept for 17 source concepts such as Read B906.00 “Neoplasm of uncertain behavior of larynx,” ICD-O-3 8000/1-C32.9 “Neoplasm, uncertain whether benign or malignant of the larynx, NOS,” ICD-9-CM 235.6 “Neoplasm of uncertain behavior of larynx,” and others. One-to-one mapping relationships are the next most common group. This is a common occurrence in the Device and Observation domains as they lack strong harmonization into standard concepts. One-to-many relationships are relatively uncommon in most domains and usually reflect exaggerated pre-coordination in source concepts. For example, ICD-10 code M05.132 “Rheumatoid lung disease with rheumatoid arthritis of left wrist” is a concept combining 2 individual meanings into one and therefore maps to 2 SNOMED-CT concepts, 1073751000119106 “Rheumatoid arthritis of left wrist” and 319841000119107 “Rheumatoid lung disease with rheumatoid arthritis,” respectively. In the Measurement domain, one-to-many equivalence relationships are due to splitting genetic variants into their genomic, transcript, and protein manifestations. The Measurement domain also has many-to-many relationships, collecting multiple source concepts into one and splitting some of them up.

**Table 2.** Distribution of equivalence relationships per type and domain.

	Type of “Maps to” relationship, % (*n*)			
Domain	One-to-one	Many-to-one	One-to-many	Many-to-many
Condition	1.9% (54 671)	10.3% (292 507)	<0.1% (38)	3.2% (90 034)
Device	6.1% (172 216)	3.6% (101 774)	<0.1% (4)	<0.1% (10)
Drug	18.1% (515 360)	42.6% (1 208 579)	<0.1% (181)	1.6% (44 780)
Measurement	0.5% (14 502)	0.4% (11 580)	1.2% (33 655)	1.2% (33 489)
Observation	3% (86 014)	2.4% (69458)	<0.1% (3)	0.2% (4579)
Procedure	1.1% (29 973)	2.5% (70 974)	<0.1% (16)	0.2% (5581)

### Release process and distribution

The OHDSI Standardized Vocabularies are released semiannually, including both source updates and OHDSI-driven modifications. Typically, the concepts and relationships between 2 releases do not differ substantially, allowing interoperability even when data sources are on different versions of the system. However, sometimes OHDSI Working Groups issue new or substantially revised content in their area of interest, such as in oncology, genetic data, and vaccines.

Since the introduction of ATHENA in 2015 as a tool for browsing and downloading of the Vocabularies, a total of more than 8600 users have downloaded a total of more than 50 000 releases.

## Discussion

Since the first postulation of the principles of a biomedical ontology^10^^,^^30^ as a mechanism of machine-processable descriptions of scientific domains and the integration of disparate data sources, these ontology systems have enabled data aggregation, vastly improved search,^31^ and allowed the statistical inference of new associations. We believe that even though these systems narrowed the semantic space or led to dimensionality reduction,^32^ when used on their own they still fall short of the goal of addressing the challenges of research in a distributed data network. We believe the OMOP CDM in conjunction with the OHDSI Standardized Vocabularies can standardize the data and their context with the required rigor to allow scalable federated research applications. These have resulted in numerous network studies, some of them of very large scale, such as the Large-Scale Evidence Generation and Evaluation in a Network of Databases (LEGEND) for studying treatments of hypertension and depression, or the Your Baseline Disease In SARS-COV-2 (CHARYBDIS) study.^33–46^

Since its inception in early 2009, the Standardized Vocabularies have grown to a proportion that is only matched by the UMLS, starting with initially 22 vocabularies to now 136. Despite that growth, it successfully kept its content consistent, so that the original OMOP experiment of 2009^29^ could be reproduced today.

However, standardizing vocabularies is an endeavor that will never conclude. Concepts and terms are constantly added, corrected, split, and combined; mistakes are identified and fixed; and relationships are overhauled. Even though the core of this resource is stable, on the fringes, there is constant movement. That creates the potential of errors: mappings may be erroneous, concept standardization may miss some semantic redundancy, and clinical events may be stored in unsupported vocabularies. For example, details of tumor attributes and genomic data are increasingly relevant to oncology research, but no vocabularies with sufficient coverage of these data elements are available. Two OHDSI Working Groups are currently addressing these shortcomings, adding vocabularies, concepts, and relationships to the Standardized Vocabularies. Nevertheless, the overall system can be considered robust as validation experiments comparing analytical methods in OMOP CDM with their native source structure so far detected only minimal effects on the overall conclusions.^11^^,^^47^^,^^48^

Another challenge stems from the need to create one standard representation for each semantic entity. To achieve that, concepts need to be mapped to each other, a complex and time-consuming process also known as ontology alignment. While the UMLS creates such crosswalks between vocabularies, to our knowledge, the OHDSI Standardized Vocabularies is the only entity that aims to achieve this comprehensively, unambiguously, United States, and non-United States. This is an ongoing process, and the maturity of semantic standardization varies highly between domains. For some, such as Drug, it could only be achieved by creating new vocabularies (RxNorm Extension),^49^ since RxNorm provides the drug formulations for the United States only and no other consolidated public source exists for international markets. For others, such as Procedure domain, standardization is confounded by the presence of concepts with different granularities so that no single ontology can be selected as a standard and a multi-ontology polyhierarchy is required instead.

Complex systems like that require a Quality Management System, that is, a formalized approach with documented processes, procedures, and responsibilities for achieving stated policies and objectives. It achieves these quality objectives through quality planning, quality assurance, quality control, and quality improvement.^50^ Such a system will require specific and quantitative assessment to be disseminated to the public. The quality standards should be defined at each level, including the effect of semantic standardization on the reliability of observational research. More needs to be done to arrive at such a maturity level.

We do not curate lateral or semantic relationships between concepts, but instead import them together with the vocabularies if available. For example, SNOMED-CT therapeutic procedures are linked to their indications using the “Has focus of” relationships. If such links were available at a comprehensive level and high quality, they could be used in lieu of a medical knowledgebase in automated queries. However, we believe achieving this system-wide is probably infeasible and less pressing for our use cases: OHDSI conducts its research to estimate relationships of that kind (eg, associations between drugs and outcomes), rather than to collect the world’s knowledge about them.

## Conclusions

The OHDSI Standardized Vocabularies are a mature and reliable resource to power the world’s largest distributed data network. It enables the application of standardized large-scale analytical methods in a truly federated setting, leading to the generation of relevant findings and publications in the field of observational research.

## Supplementary Material

ocad247_Supplementary_Data

## Data Availability

All vocabulary data can be accessed through Athena (https://athena.ohdsi.org/vocabulary/list). Most vocabularies can be downloaded for free. Vocabularies requiring an End User License Agreement are distributed upon proof of license with the authoring organization.
