# Supplementary material for: OHDSI Standardized Vocabularies—a large-scale centralized reference ontology for international data harmonization
Source: J Am Med Inform Assoc. 2024 Jan 4;31(3):583–90. doi: 10.1093/jamia/ocad247 (PMC10873827; doi:10.1093/jamia/ocad247)
Supplement: ocad247_Supplementary_Data [file ocad247_supplementary_data.docx]

**Supplementary Table 1.** Vocabularies incorporated into the OMOP Standardized Vocabularies with country of origin, status (OMOP generated, imported or imported and mapped) and volume (valid concepts and relationships).

| **Vocabulary id** | **Vocabulary name** | **Country of origin** | **Status** | **Sourced from** | **Volume** |
| --- | --- | --- | --- | --- | --- |
| ABMS | Provider Specialty (American Board of Medical Specialties) | USA | Imported | Source | 98 concepts, 490 relationships |
| AMIS | Medicinal Products Information System (DIMDI) | Germany | Imported and mapped | Source | 142066 concepts, 121552 relationships |
| AMT | Australian Medicines Terminology (NEHTA) | Australia | Imported and mapped | Source | 136850 concepts, 142575 relationships |
| APC | Ambulatory Payment Classification (CMS) | USA | Imported | Source | 1910 concepts, 1430 relationships |
| ATC | WHO Anatomic Therapeutic Chemical Classification | International | Imported and mapped | UMLS | 6740 concepts, 262406 relationships |
| BDPM | Public Database of Medications (Social-Sante) | France | Imported and mapped | Source | 44376 concepts, 47390 relationships |
| Cancer Modifier | Diagnostic Modifiers of Cancer (OMOP) |  | OMOP generated |  | 6043 concepts, 33473 relationships |
| CAP | CAP electronic Cancer Checklists (College of American Pathologists) | USA | Imported | Source | 18464 concepts, 111490 relationships |
| CCAM | Common Classification of Medical Acts | France | Imported | Source | 10206 concepts, 47636 relationships |
| CDT | Current Dental Terminology (ADA) | USA | Imported | UMLS | 926 concepts, 2284 relationships |
| CGI | Cancer Genome Interpreter (Pompeu Fabra University) | Spain | Imported and mapped | Source | 5351 concepts, 550 relationships |
| CIEL | Columbia International eHealth Laboratory (Columbia University) | USA | Imported | Source | 50881 concepts, 44394 relationships |
| CIM10 | International Classification of Diseases, Tenth Revision, French Edition | France | Imported and mapped | Source | 12226 concepts, 35888 relationships |
| CIViC | Clinical Interpretation of Variants in Cancer (civicdb.org) | USA | Imported | Source | 1386 concepts, 2557 relationships |
| ClinVar | ClinVar (NCBI) | USA | Imported | Source | 8072 concepts, 47090 relationships |
| CMS Place of Service | Place of Service Codes for Professional Claims (CMS) | USA | Imported | Source | 60 concepts, 657 relationships |
| Cohort | Legacy OMOP HOI or DOI cohort |  | OMOP generated |  | 78 concepts, 2742 relationships |
| Concept Class | OMOP Concept Class |  | OMOP generated |  | 416 concepts, |
| Condition Status | OMOP Condition Status |  | OMOP generated |  | 22 concepts, 76 relationships |
| Condition Type | OMOP Condition Occurrence Type |  | OMOP generated |  | 118 concepts |
| COSMIC | Catalogue Of Somatic Mutations In Cancer | UK | Imported | Source | 273604 concepts |
| Cost | OMOP Cost |  | OMOP generated |  | 51 concepts, 102 relatiosnhips |
| Cost Type | OMOP Cost Type |  | OMOP generated |  | 8 concepts |
| CPT4 | Current Procedural Terminology version 4 (AMA) | USA | Imported and mapped | UMLS | 16922 concepts, 92782 relationships |
| CTD | Comparative Toxicogenomic Database | USA | Imported and mapped | Source | 8698 concepts, 8635 relationships |
| Currency | International Currency Symbol (ISO 4217) | International | Imported | Source | 180 concepts, 360 relationships |
| CVX | CDC Vaccine Administered CVX (NCIRD) | USA | Imported and mapped | Source | 251 concepts, 3990 relationships |
| DA_France | Disease Analyzer France (IQVIA) | France | Imported and mapped | Source | 144783 concepts, 76805 relationships |
| Death Type | OMOP Death Type |  | OMOP generated |  | 14 concepts |
| Device Type | OMOP Device Type |  | OMOP generated |  | 4 concepts |
| dm+d | Dictionary of Medicines and Devices (NHS) | UK | Imported and mapped | Source | 387449 concepts, 417041 relationships |
| Domain | OMOP Domain |  | OMOP generated | Source | 65 concepts, 22 relationships |
| DPD | Drug Product Database (Health Canada) | Canada | Imported and mapped | Source | 193647 concepts, 50078 relationships |
| DRG | Diagnosis-related group (CMS) | USA | Imported | Source | 1362 concepts, 3695 relationships |
| Drug Type | OMOP Drug Exposure Type |  | OMOP generated |  | 16 concepts |
| EDI | Korean EDI | Korea | Imported and mapped | Source | 313431 concepts, 2500 relationships |
| EphMRA ATC | Anatomical Classification of Pharmaceutical Products (EphMRA) | International | Imported and mapped | Source | 895 concepts, 1758 relationships |
| Episode | OMOP Episode |  | OMOP generated |  | 18 concepts, 47 relationships |
| Episode Type | OMOP Episode Type |  | OMOP generated |  | 5 concepts |
| ETC | Enhanced Therapeutic Classification (FDB) | USA | Imported | UMLS | 2780 concepts, 43418 relationships |
| Ethnicity | OMOP Ethnicity |  | OMOP generated |  | 2 concepts, 6 relationships |
| GCN_SEQNO | Clinical Formulation ID (FDB) | USA | Imported | Source | 29659 concepts, 14460 relationships |
| Gemscript | Gemscript (Resip) | UK | Imported and mapped | Source | 260517 concepts, 326328 relationships |
| Gender | OMOP Gender |  | OMOP generated |  | 5 concepts, 9 relationships |
| GGR | Commented Drug Directory (BCFI) | Belgium | Imported and mapped | Source | 27208 concepts, 27087 relationships |
| GPI | Medi-Span Generic Product Identifier (Wolters Kluwer Health) | USA | Imported | Source | 36764 concepts, 20967 relationships |
| GRR | Global Reference Repository (IQVIA) | Germany | Imported and mapped | Source | 707386 concepts, 1022785 relationships |
| HCPCS | Healthcare Common Procedure Coding System (CMS) | USA | Imported and mapped | UMLS | 11269 concepts, 22002 relationships |
| HemOnc | HemOnc | USA | Imported | Source | 8028 concepts, 80949 relationships |
| HES Specialty | Hospital Episode Statistics Specialty (NHS) | UK | Imported | Source | 165 concepts, 365 relationships |
| HGNC | Human Gene Nomenclature (European Bioinformatics Institute) | International | Imported | Source | 16519 concepts, 56861 relationships |
| ICD10 | International Classification of Diseases, Tenth Revision (WHO) | International | Imported and mapped | Source | 98583 concepts, 703812 relationships |
| ICD10CM | International Classification of Diseases, Tenth Revision, Clinical Modification (NCHS) | International | Imported and mapped | Source | 34491 concepts, 274209 relationships |
| ICD10CN | International Classification of Diseases, Tenth Revision, Chinese Edition | China | Imported and mapped | Source | 17213 concepts, 62336 relationships |
| ICD10GM | International Classification of Diseases, Tenth Revision, German Edition | Germany | Imported and mapped | Source | 194981 concepts, 1345032 relationships |
| ICD10PCS | ICD-10 Procedure Coding System (CMS) | USA | Imported and mapped | UMLS | 17564 concepts, 71543 relationships |
| ICD9CM | International Classification of Diseases, Ninth Revision, Clinical Modification, Volume 1 and 2 (NCHS) | USA | Imported and mapped | UMLS | 4657 concepts, 31907 relationships |
| ICD9Proc | International Classification of Diseases, Ninth Revision, Clinical Modification, Volume 3 (NCHS) | International | Imported and mapped | Source | 13385 concepts, 40013 relationships |
| ICD9ProcCN | International Classification of Diseases, Ninth Revision, Chinese Edition, Procedures | China | Imported and mapped | Source | 64471 concepts, 779357 relationships |
| ICDO3 | International Classification of Diseases for Oncology, Third Edition (WHO) | International | Imported and mapped | Source | 4863 concepts, 403340 relationships |
| Indication | Indications and Contraindications (FDB) | USA | Imported | Source | 17336 concepts, 135198 relationships |
| ISBT | Information Standard for Blood and Transplant 128 Product (ICCBBA) | USA | Imported | Source | 1657 concepts, 102818 relationships |
| ISBT Attribute | Information Standard for Blood and Transplant 128 Product Attribute (ICCBBA) | USA | Imported | Source | 7855 concepts, 18064 relationships |
| JAX | The Clinical Knowledgebase (The Jackson Laboratory) | USA | Imported | Source | 38798 concepts, 40498 relationships |
| JMDC | Japan Medical Data Center Drug Code (JMDC) | Japan | Imported and mapped | Source | 22508 concepts, 78542 relationships |
| KCD7 | Korean Classification of Diseases, 7th Revision | Korea | Imported and mapped | Source | 63861 concepts, 64382 relationships |
| KDC | Korean Drug Code (HIRA) | Korea | Imported and mapped | Source | 3 concepts, 6 relationships |
| KNHIS | Korean National Health Information System | Korea | Imported | Source | 7 concepts, 14 relationships |
| Korean Revenue Code | Korean Revenue Code | Korea | Imported | Source | 1 concepts, 3 relationships |
| LOINC | Logical Observation Identifiers Names and Codes (Regenstrief Institute) | USA | Imported | Source | 265076 concepts, 2255835 relationships |
| LPD_Australia | Longitudinal Patient Data Australia (IQVIA) | Australia | Imported and mapped | Source | 35773 concepts, 31227 relationships |
| LPD_Belgium | Longitudinal Patient Data Belgium (IQVIA) | Belgium | Imported and mapped | Source | 35490 concepts, 20255 relationships |
| MDC | Major Diagnostic Categories (CMS) | USA | Imported | Source | 26 concepts, 1351 relationships |
| Meas Type | OMOP Measurement Type |  | OMOP generated |  | 12 concepts |
| MedDRA | Medical Dictionary for Regulatory Activities (MSSO) | USA | Imported | Source | 108301 concepts, 273229 relationships |
| Medicare Specialty | Medicare provider/supplier specialty codes (CMS) | USA | Imported | Source | 120 concepts, 1157 relationships |
| MeSH | Medical Subject Headings (NLM) | USA | Imported | UMLS | 14118 concepts, 13739 relationships |
| Metadata | Metadata |  | OMOP generated |  | 2 concepts, 317 relationships |
| MMI | Modernizing Medicine (MMI) | USA | Imported | Source | 4 concepts, 8 relationships |
| Multilex | Multilex (FDB) | USA | Imported | Source | 77177 concepts, 193354 relationships |
| Multum | Cerner Multum (Cerner) | USA | Imported | Source | 9770 concepts, 8723 relationships |
| NAACCR | Data Standards & Data Dictionary Volume II (NAACCR) | USA | Imported | Source | 34473 concepts, 1562445 relationships |
| NCCD | Normalized Chinese Clinical Drug | China | Imported and mapped | Source | 51583 concepts, 51609 relationships |
| NCIt | NCI Thesaurus (National Cancer Institute) | USA | Imported | Source | 2426 concepts, 20952 relationships |
| NDC | National Drug Code (FDA and manufacturers) | USA | Imported | Source | 1147408 concepts, 910878 relationships |
| NDFRT | National Drug File - Reference Terminology (VA) | USA | Imported | Source | 69567 concepts, 798332 relationships |
| Nebraska Lexicon | Nebraska Lexicon | USA | Imported | Source | 465801 concepts, 2827961 relationships |
| NFC | New Form Code (EphMRA) | International | Imported | Source | 692 concepts, 1348 relationships |
| Note Type | OMOP Note Type |  | OMOP generated |  | 10 concepts |
| NUCC | National Uniform Claim Committee Health Care Provider Taxonomy Code Set (NUCC) | USA | Imported | Source | 855 concepts, 2826 relationships |
| Obs Period Type | OMOP Observation Period Type |  | OMOP generated |  | 6 concepts |
| Observation Type | OMOP Observation Type |  | OMOP generated |  | 29 concepts |
| OMOP Extension | OMOP Extension (OHDSI) |  | OMOP generated |  | 1240 concepts, 11093 relationships |
| OMOP Genomic | OMOP Genomic vocabulary |  | OMOP generated |  | 120991 concepts, 549693 relationships |
| OMOP Invest Drug | OMOP Investigational Drugs | USA | Imported and mapped | Source | 29727 concepts, 46573 relationships |
| OncoKB | Oncology Knowledge Base (MSK) | USA | Imported | Source | 5569 concepts, 4708 relationships |
| OncoTree | OncoTree (MSK) | USA | Imported and mapped | Source | 885 concepts, 3509 relationships |
| OPCS4 | OPCS Classification of Interventions and Procedures version 4 (NHS) | UK | Imported and mapped | Source | 11000 concepts, 21921 relationships |
| OPS | Operations and Procedures Classification (OPS) | Germany | Imported | Source | 42959 concepts, 95389 relationships |
| OSM | OpenStreetMap | UK | Imported | Source | 203339 concepts, 813271 relationships |
| OXMIS | Oxford Medical Information System (OCHP) | UK | Imported and mapped | Source | 8118 concepts, 7895 relationships |
| PCORNet | National Patient-Centered Clinical Research Network (PCORI) | USA | Imported | Source | 81 concepts, 52 relationships |
| Plan | Health Plan - contract to administer healthcare transactions by the payer, facilitated by the sponsor |  | OMOP generated |  | 11 concepts, 22 relationships |
| Plan Stop Reason | Plan Stop Reason - Reason for termination of the Health Plan |  | OMOP generated |  | 13 concepts, 26 relationships |
| PPI | AllOfUs_PPI (Columbia) | USA | Imported | Source | 6529 concepts, 48062 relationships |
| Procedure Type | OMOP Procedure Occurrence Type |  | OMOP generated |  | 97 concepts |
| Provider | OMOP Provider |  | OMOP generated |  | 6 concepts, 245 relationships |
| Race | Race and Ethnicity Code Set (USBC) | USA | Imported | Source | 53 concepts, 723 relationships |
| Read | NHS UK Read Codes Version 2 (HSCIC) | UK | Imported and mapped | Source | 108945 concepts, 109807 relationships |
| Relationship | OMOP Relationship |  | OMOP generated |  | 712 concepts, 36 relationships |
| Revenue Code | UB04/CMS1450 Revenue Codes (CMS) | USA | Imported | Source | 538 concepts, 1076 relationships |
| RxNorm | RxNorm (NLM) | USA | Imported | UMLS | 304866 concepts, 7483100 relationships |
| RxNorm Extension | RxNorm Extension (OHDSI) | non-US | OMOP generated | Source | 2110429 concepts, 18993925 relationships |
| SMQ | Standardised MedDRA Queries (MSSO) | USA | Imported | Source | 324 concepts, 16985 relationships |
| SNOMED | Systematic Nomenclature of Medicine - Clinical Terms (IHTSDO) | International | Imported and mapped | UMLS | 1054935 concepts, 18239577 relationships |
| SNOMED Veterinary | SNOMED Veterinary | International | Imported | Source | 33684 concepts, 137590 relationships |
| SOPT | Source of Payment Typology (PHDSC) | USA | Imported | Source | 168 concepts, 644 relationships |
| Specimen Type | OMOP Specimen Type |  | OMOP generated |  | 1 concept |
| SPL | Structured Product Labeling (FDA) | USA | Imported | Source | 652709 concepts, 708743 relationships |
| Sponsor | Sponsor - institution or individual financing healthcare transactions |  | OMOP generated |  | 6 concepts, 12 relationships |
| Supplier | OMOP Supplier |  | OMOP generated |  | 1 concept |
| SUS | Table of Procedures, Drugs, Orthoses, Protheses and Special Materials (Brazilian Unified Health System) | Brazil | Imported and mapped | Source | 4593 concepts, 4663 relationships |
| Type Concept | OMOP Type Concept |  | OMOP generated |  | 80 concepts, 370 relationships |
| UB04 Point of Origin | UB04 Claim Source Inpatient Admission Code (CMS) | USA | Imported | Source | 23 concepts, 13 relationships |
| UB04 Pri Typ of Adm | UB04 Claim Inpatient Admission Type Code (CMS) | USA | Imported | Source | 6 concepts, 12 relationships |
| UB04 Pt dis status | UB04 Patient Discharge Status Code (CMS) | USA | Imported | Source | 55 concepts, 41 relationships |
| UB04 Typ bill | UB04 Type of Bill - Institutional (USHIK) | USA | Imported | Source | 298 concepts, 321 relationships |
| UCUM | Unified Code for Units of Measure (Regenstrief Institute) | USA | Imported | Source | 1118 concepts, 6703 relationships |
| UK Biobank | UK Biobank | UK | Imported and mapped | Source | 19337 concepts, 77807 relationships |
| US Census | United States Census Bureau | USA | Imported | Source | 13 concepts, 99 relationships |
| VA Class | VA National Drug File Class (VA) | USA | Imported | Source | 576 concepts, 50998 relationships |
| VANDF | VA National Drug File Product (VA) | USA | Imported and mapped | UMLS | 40136 concepts, 50998 relationships |
| Visit | OMOP Visit |  | OMOP generated |  | 19 concepts, 397 relationships |
| Visit Type | OMOP Visit Type |  | OMOP generated |  | 18 concepts, 30 relationships |
| Vocabulary | OMOP Vocabulary |  | OMOP generated |  | 143 concepts |

**Supplementary Table 2.** Main domains in OMOP Standardized Vocabularies with corresponding target OMOP CDM tables along with the Vocabularies within the domains

| **Domain** | **Table** | **Vocabulary Category** | | | |
| --- | --- | --- | --- | --- | --- |
|  |  | **Preferred for standard** | **Classification** | **Source** | **Preferred for specific case** |
| Condition | CONDITION_OCCURRENCE | SNOMED, OMOP Extension | MedDRA, SMQ | ICD-10, ICD-10(CM), ICD-10(CN), ICD-10(GM), ICD-9(CM), KCD7, CIM10, CIEL, HemOnc, OPCS4, OXMIS, PPI, Read | Nebraska Lexicon, ICDO3 for oncology; SNOMED Veterinary for veterinary |
| Device | DEVICE_EXPOSURE | SNOMED, HCPCS, CPT4, ICD10PCS, CDT* | SPL | AMIS, DA_France, EDI, dm+d, HCPCS, CIEL, OXMIS, OPCS4, Nebraska Lexicon, Gemscript, LPD_Australia, Read, SUS, NCCD, LPD_Belgium, GGR, ICD10PCS, ISBT, JMDC, KDC, dm+d, NDC, AMT, OPCS4 | SNOMED Veterinary for veterinary |
| Drug | DRUG_EXPOSURE | RxNorm, RxNorm Extension, CVX | ATC, EphMRA ATC, ETC, NDFRT, CVX, Indication, SPL | AMIS, LPD_Belgium, DA_France, GGR, ICD10PCS, VA Product, SNOMED Veterinary, Multilex, EDI, dm+d, JMDC, NAACCR, KDC, DPD, SNOMED, NDC, HCPCS, CPT4, Multum, CTD, CIEL, OXMIS, HemOnc, GPI, AMT, VA Class, Nebraska Lexicon, Gemscript, BDPM, PPI, LPD_Australia, GRR, Read, GCN_SEQNO, MeSH, SUS, NCCD | CVX for vaccine exposure |
| Measurement | MEASUREMENT | LOINC, SNOMED, OMOP Extension* | LOINC, MeDRA | ICD-10(CM), CAP, CGI, EDI, NAACCR, JAX, ICD-10, HCPCS, CPT4, CIEL, OXMIS, ClinVar, OPCS4, Nebraska Lexicon, PPI, ICD-10(GM), NCIt, OncoKB, Read, MeSH, ICD-9(CM), SUS, KCD7, ICD-10(CN), CIM10, CIViC, UK Biobank | PPI for surveys |
| Observation | OBSERVATION | SNOMED, HCPCS, CPT4, LOINC, OMOP Extension* | LOINC, MedDRA | ICD-10(CM), CAP, NAACCR, ICD-10, HCPCS, CIEL, OXMIS, LOINC, PCORNet, OPCS4, Nebraska Lexicon, PPI, ICD-10(GM), Read, ICD-9(CM), SUS, KCD7, ICD-10CN, CIM10, UK Biobank, ICDO3 | SNOMED Veterinary for veterinary, PPI for surveys, Nebraska Lexicon and NAACCR for oncology |
| Procedure | PROCEDURE_OCCURRENCE | CPT4, SNOMED, ICD10PCS, ICD9Proc, HCPCS | CPT4, MedDRA | ICD-10(CM), CCAM, EDI, NAACCR, ICD-10, OPS, CIEL, OXMIS, HemOnc, OPCS4, Nebraska Lexicon, ICD-10(GM), Read, CDT, MeSH, ICD-9(CM), MedDRA, SUS, KCD7, ICD9ProcCN, ICD-10(CN), CIM10 |  |

**Supplementary Table 3.** Distribution of concepts across six main domains stratified by concept type.

| **Domain** | **Concept type** | **Number of valid concepts (%)** |
| --- | --- | --- |
| Condition | Classification | 56,010 (0.67%) |
|  | Standard | 173,625 (2.07%) |
|  | Non-standard | 405,606 (4.83%) |
| Device | Classification | 1,815 (0.02%) |
|  | Standard | 479,971 (5.72%) |
|  | Non-standard | 314,109 (3.74%) |
| Drug | Classification | 687,600 (8.20%) |
|  | Standard | 1,984,719 (23.66%) |
|  | Non-standard | 2,613,154 (31.155%) |
| Measurement | Classification | 56,958 (0.68%) |
|  | Standard | 173,303 (2.07%) |
|  | Non-standard | 349,432 (4.17%) |
| Observation | Classification | 5,203 (0.06%) |
|  | Standard | 173,092 (2.06%) |
|  | Non-standard | 238,717 (2.85%) |
| Procedure | Classification | 1,4037 (0.17%) |
|  | Standard | 268,640 (3.20%) |
|  | Non-standard | 393,508 (4.70%) |

**Supplementary Table 4.** Distribution of top relationships in the OMOP Standardized Vocabularies.

| **Relationship type** | **Number of valid relationships (%)** |
| --- | --- |
| Is a/Subsumes | 4,207,033 (14.85%) |
| Maps to/Mapped from | 3,986,653 (14.07%) |
| Has marketed form/Marketed form of | 1,941,193 (6.85%) |
| RxNorm has dose form/RxNorm dose form of | 1,762,492 (6.22%) |
| Has brand name/Brand name of | 1,451,998 (5.13%) |
| Has module/Module of | 1,057,008 (3.73%) |
| Has status/Status of | 1,054,576 (3.73%) |
| Tradename of/Has tradename | 1,046,554 (3.70%) |
| RxNorm - SPL/SPL - RxNorm | 656,905 (2.31%) |
| Has supplier/Supplier of | 557,946 (1.97%) |
| Available as box/Box of | 545,166 (1.92%) |
| RxNorm is a/RxNorm inverse is a | 476,747 (1.68%) |
| Constitutes/Consists of | 432,805 (1.53%) |
| Concept replaces/Concept replaced by | 388,844 (1.37%) |
| Has non-avail ind/Non-avail ind of | 363,283 (1.28%) |
| VMP has prescr stat/VMP prescr stat of | 363,161 (1.28%) |
| Has quantified form/Quantified form of | 362,118 (1.28%) |
| Has CD category/CD category of | 359,400 (1.27%) |
| Has AMP restr ind/AMP restr ind of | 305,409 (1.08%) |
| Has method/Method of | 285,425 (1.01%) |
